# Protective Efficacy of Decreasing Antigen Doses of a *Chlamydia abortus* Subcellular Vaccine Against Ovine Enzootic Abortion in a Pregnant Sheep Challenge Model

**DOI:** 10.3390/vaccines13010089

**Published:** 2025-01-18

**Authors:** Morag Livingstone, Kevin Aitchison, Javier Palarea-Albaladejo, Francesco Ciampi, Clare Underwood, Antonia Paladino, Francesca Chianini, Gary Entrican, Sean Ranjan Wattegedera, David Longbottom

**Affiliations:** 1 Moredun Research Institute, Pentlands Science Park, Penicuik, Midlothian EH26 0PZ, UK; morag.livingstone@moredun.ac.uk (M.L.); kevin.aitchison@moredun.ac.uk (K.A.); ciampi@bonassisa.it (F.C.); clare.underwood@moredun.ac.uk (C.U.); antonia.paladino@uslsudest.toscana.it (A.P.); fchianini@yahoo.com (F.C.); gary.entrican@roslin.ed.ac.uk (G.E.); sean.wattegedera@moredun.ac.uk (S.R.W.); 2 Biomathematics and Statistics Scotland, Edinburgh EH9 3FD, UK; javier.palarea@udg.edu

**Keywords:** *Chlamydia abortus*, ovine enzootic abortion, vaccine development, vaccine efficacy, quantitative real-time PCR, serological analysis, cytokine analysis

## Abstract

Background/Objective: *Chlamydia abortus*, the cause of ovine enzootic abortion, is a zoonotic bacterial pathogen and one of the most infectious causes of foetal death in sheep worldwide. Although the disease can be controlled using commercial inactivated and live whole-organism vaccines, there are issues with both, particularly concerning efficacy and safety. Recently, we have described the development of a new COMC (chlamydial outer membrane complex) vaccine based on a detergent-extracted outer membrane protein preparation of the pathogen, which can be delivered in a single inoculation and is both efficacious and safe. Methods: In this study, we have evaluated the COMC vaccine further in a dose–response titration of the chlamydial antigen content of the vaccine (from 20 to 2.5 µg in seven experimental groups) using an established pregnant sheep challenge model. Results: No obvious dose–response relationship was observed across the groups, with a single abortion event occurring in four of the groups and three in the lowest dose group (2.5 µg). No abortions occurred in the 15 and 10 µg groups. The abortion rates (0–14%) were significantly below that of the challenge control group (33%). A similar reduction in bacterial shedding of infectious organisms following parturition was observed in the vaccinated groups compared to the challenge control group, which is important in terms of reducing potential transmission to naive animals. Conclusions: The results show that a dose of 10 µg antigen in the vaccine will be optimal in terms of maximising efficacy, reducing shedding at parturition, and ensuring it is cost-effective to produce for commercial manufacture.

## 1. Introduction

Chlamydial abortion in sheep (syn: ovine enzootic abortion (OEA); enzootic abortion of ewes (EAE); ovine chlamydiosis), first described in 1950 [1], is caused by the Gram-negative obligate intracellular bacterium *Chlamydia abortus* (*C. abortus*). The pathogen is a common cause of infectious abortion in livestock worldwide, where it predominantly infects sheep and goats, resulting in significant economic losses, and can also infect other animal species, including cattle, pigs, and horses [2,3,4,5,6]. *Chlamydia abortus* is also a zoonotic pathogen and presents a risk to immunocompromised individuals and can also cause abortion and life-threatening illness in pregnant women [7,8,9,10,11].

In *C. abortus*-infected flocks, the disease affects the placenta, causing a typical necrotising placentitis and vasculitis, often accompanied by a reddish or dirty pink exudate, and resulting in abortion or stillbirths towards the end of pregnancy, usually within the last two to three weeks [1]. Generally, there is no indication that an abortion, which is commonly accompanied by the birth of weak or healthy live lambs, is going to occur [12]. The disease is thought to spread to naïve animals via the oro-nasal route at lambing time where infected ewes shed large amounts of *C. abortus* in infected placentas and vaginal discharges at abortion or parturition [2,3,13]. In non-pregnant animals, *C. abortus* remains in a latent persistent state until the onset of pregnancy when there is recrudescence and rapid multiplication of the pathogen, initially in the trophoblast cells of the cotyledon which spreads to the surrounding chorion, leading to the destruction of the placental tissue and eventual abortion [14]. Following abortion, ewes are immune from further abortive episodes; however, they may still shed infectious organisms in subsequent pregnancies, thereby posing a risk of infection to other animals.

Vaccination is still the most effective way of protecting sheep from abortion and preventing zoonotic infection in humans [15,16,17,18,19,20,21]. In the UK, there are currently three vaccines available to protect against the disease; two are live-attenuated vaccines utilising the French 1B strain of *C. abortus* [20,22] and the third is an inactivated vaccine based on the UK A22 strain of *C. abortus* [23] that has been combined with *Salmonella enterica* subsp. *enterica* serovar Abortusovis [15]. This A22 strain was incorporated in the very first commercial inactivated *C. abortus* vaccine, which was developed by Moredun in the 1950s [24,25,26] in conjunction with the Wellcome Foundation, and had some success [23], but eventually, it was withdrawn from the market in 1992 due to loss of efficacy [16,21,27,28]. The lower efficacy of inactivated and recombinant-based vaccines [15,29,30,31,32], as well as other issues, particularly safety concerns with the live vaccines being shown to cause infections and disease in some animals [33,34], have led us to develop a safer efficacious subcellular vaccine [35] based on the *C. abortus* chlamydial outer membrane complex (COMC) [36], which largely comprises the major outer membrane protein (MOMP) [37] and has been previously shown to be protective against other chlamydial species in a number of animal vaccine efficacy studies [38,39,40].

In our previous study, we determined that the *C. abortus* COMC vaccine can be delivered in a lower dose as a single inoculation without affecting its efficacy [41]. This present study aimed to explore whether the antigen dose in the vaccine could be reduced even further in order to keep the manufacturing costs of a commercial vaccine as low as possible for the veterinary market. This was achieved through a dose–response study using our well-established pregnant sheep challenge model [35,41,42,43,44,45,46] and assessing vaccine efficacy through a reduction in adverse pregnancy outcomes, placental pathology, and organism shedding in vaginal fluids following parturition.

## 2. Materials and Methods

### 2.1. Ethics Statement

The experimental protocol was approved by the Moredun Animal Welfare and Ethical Review Body (permit number: E27/15; 22 June 2015). All husbandry practices and animal procedures were carried out in strict accordance with the Animals (Scientific Procedures) Act 1986, as well as in compliance with all UK Home Office Inspectorate regulations and ARRIVE guidelines 2.0 [47]. Animals were monitored at least three times daily throughout the duration of the study for any clinical signs or welfare issues. This monitoring was increased to 24 h per day in the last four weeks of expected parturition. Any animal found to be suffering or requiring treatment was given appropriate veterinary care, which included, if necessary, the use of antibiotics. Any weak or non-viable lambs born in the experimental and challenge control groups were assessed and monitored by a registered veterinary practitioner and were euthanised by administering an overdose of 20% *w*/*v* sodium pentobarbital (Pentoject^®^, Animalcare Ltd., New York, NY, UK; #XVD133), if necessary, to end suffering. Such decisions were based on a range of criteria, including not being able to stand or lift its head (generally lying flat out on its side), not opening its eyes, not being able to show or showing no interest in suckling, exhibiting laboured respiration, and generally showing minimal signs of life. Ewes and lambs were kept under surveillance for an additional 2 months following parturition, with veterinary care and intervention where required.

### 2.2. Preparation of C. abortus Elementary Bodies and COMC Vaccine Antigen

*Chlamydia abortus* strain S26/3, which was isolated in 1979 from the placenta of a vaccinated ewe that aborted [48], was propagated in McCoy cells, as previously described [48,49]. Briefly, the strain was grown in Roswell Park Memorial Institute (RPMI) 1640 Medium (Fisher Scientific UK, Loughborough, UK, #11470315), supplemented with 2% foetal bovine serum and 1 μg/mL cycloheximide (Merck Life Science UK Ltd. (Sigma-Aldrich Co.), Gillingham, UK; #C4859), in Corning 225 cm^2^ flasks (Scientific Laboratory Supplies Ltd., Newhouse, UK; #431082) at 37 °C under 5% CO_2_ for 3 days. Infected cells were harvested from flasks using glass beads with vigorous shaking and centrifuged at 500× *g* for 5 min to remove gross cellular debris. The supernatant was centrifuged at 20,000× *g* to pellet the chlamydial elementary bodies (EBs), which were subsequently purified on discontinuous Gastrografin (Bayer, Reading, UK; #82273670) gradients, according to the method of Buendia et al. [50]. Purified EBs were suspended in 0.1 M phosphate-buffered saline (PBS), at pH 7.2, and stored at −70 °C until required.

EBs were quantified using a Pierce™ BCA Protein Assay Kit (Thermo Fisher Scientific, Paisley, UK; #23227), following solubilisation in 0.2 M sodium hydroxide for 1 h at 37 °C, according to the manufacturer’s instructions. The COMC antigen was prepared from EBs by sequential extraction in 2% sarkosyl (N-lauroylsarcosine sodium salt; Merck Life Science UK Ltd., London, UK, #61743) at 37 °C for 1 h, followed by a further 1 h incubation in 2% sarkosyl/10 mM dithiothreitol (DTT; Promega UK, Southampton, UK; #V3151) and differential centrifugation, as described previously [35,46]. The resulting pellet, comprising insoluble COMC (https://doi.org/10.1371/journal.pone.0224070.g001), was subjected to sodium dodecyl sulphate–polyacrylamide gel electrophoresis (SDS-PAGE) and the MOMP band was quantified by densitometry against a series of bovine serum albumin standards using ImageQuant TL 1D v8.1 gel analysis software (GE HealthCare, Chalfont St. Giles, UK) to give an estimate of MOMP concentration.

### 2.3. Formulation of COMC Vaccine Preparations

The COMC antigen was diluted in PBS to prepare the aqueous phase of the vaccine prior to adjuvanting with Montanide™ ISA 70 VG (Seppic SA, Paris, France) [51], according to the manufacturer’s instructions, using a ratio of adjuvant/antigen of 70/30 (weight/weight) and providing formulations containing final concentrations of 20, 14, 10, 7, 5, 3.5, and 2.5 µg equivalent of MOMP per 1 mL dose. Emulsification was performed using an Ultra Turrax homogeniser (IKA^®^-Werke GmbH & Co., Staufen im Breisgau, Germany) at a high shear rate at room temperature. Vaccines were prepared and stored at 4 °C for one month prior to administration to ensure stability.

### 2.4. Preparation of C. abortus Challenge Inoculum

*C. abortus* strain S26/3 was grown in the yolk sacs of fertilised hens’ eggs, suspended in PBS, and stored in liquid nitrogen, as previously described [52,53]. The EB titre was determined as described in Livingstone et al. [54]. Briefly, 1 mL aliquots of 10-fold serial dilutions of the yolk sac material, prepared in the RPMI medium described above, were inoculated onto McCoy cell monolayers grown on duplicate glass coverslips. After 72 h incubation at 37 °C/5% CO_2_, the coverslips were fixed in methanol for 10 min, stained with Giemsa solution R66 (Gurr™ for microscopical staining; VWR International Ltd., Lutterworth, UK; # 350865P) for 20 min, dehydrated through acetone/xylene graded solutions, and mounted in DPX (Merck Life Science UK Ltd.; #06522). Coverslips were examined by light microscopy and the number of chlamydial inclusions was counted at X40 magnification across the whole coverslip. The titre was calculated by multiplying the number of inclusions by the reciprocal dilution. Immediately before use, the challenge inoculum was removed from liquid nitrogen storage and diluted in PBS to provide 10^6^ inclusion forming units (IFUs) of *C. abortus* per 1 mL.

### 2.5. Experimental Design

**Figure 1:**
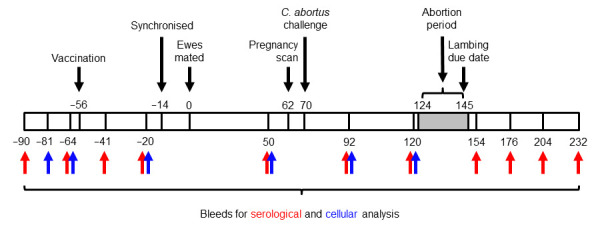
Experimental design. Numbers above and under bar indicate days prior to or post mating.

Scotch Mule sheep (crossbred Scottish Blackface ewes sired by Bluefaced Leicester rams; aged 1 to 2 years and not yet had a first lamb) were sourced commercially from EAE-accredited flocks participating in the UK Premium Sheep and Goat Health Schemes [55] and pre-screened by an rOMP90-3 enzyme-linked immunosorbent assay (ELISA) [56] and by an in vitro lymphocyte stimulation assay [57]. A total of 178 *C. abortus*-seronegative animals were selected and randomly assigned to eight groups of 21 ewes (experimental and challenge control groups) and one group of 10 (negative control group). Eight weeks prior to mating, all animals in experimental groups 1–7 received a 1 mL dose of COMC vaccine administered intramuscularly (i.m.) using a 19G 1” needle on the left side of the neck: groups 1 to 7 received a formulated vaccine containing 20, 14, 10, 7, 5, 3.5, and 2.5 µg of COMC antigen, respectively. Group 8 and 9 animals were not vaccinated and served as challenge and negative controls, respectively. Six weeks after vaccination, all ewes were synchronised using Chronogest^®^ CR 20 mg controlled-release sponges (MSD Animal Health UK Ltd., Milton Keynes, UK) over two weeks and then mated. At day 70 of gestation, all pregnant vaccinated ewes (groups 1–7) and group 8 challenge control ewes were inoculated subcutaneously (s.c.), using a 19G 1” needle, over the left prefemoral lymph node with 2 mL of challenge inoculum containing 2 × 10^6^ IFUs of *C. abortus*. Group 9 animals (unvaccinated and non-challenged negative controls) were housed remotely from the other groups. Figure 1 depicts a summary of the experimental design.

All animals received a normal maintenance diet, which included as much access to hay and water as they wanted. The clinical outcome of each ewe, as well as the weight and sex of each live or dead lamb immediately following delivery, was recorded. For the purposes of all calculations and statistical comparisons, a ewe was considered to have aborted if it delivered at least one dead lamb or a weak non-viable lamb that had to be euthanised on animal welfare grounds or which died within 48 h of birth. The cause of abortions, stillbirths, and neonatal deaths were confirmed as due to *C. abortus* if chlamydial EBs and/or DNA could be detected in the placental samples, foetal organs, or uterine discharges by mZN-stained smears, quantitative real-time polymerase chain reaction (qPCR), or through pathological investigation.

### 2.6. Sample Collection and Processing

Placentas were collected following lambing or abortion and examined for evidence of typical OEA lesions. Placentas were cleared of any attached bedding and oriented to expose the cotyledons, and the percentage of area affected by gross pathology was recorded, as previously described [44]. Where possible, two affected cotyledons plus surrounding intercotyledonary membranes were excised using sterile instruments; otherwise, one or two unaffected cotyledons were sampled. One-half of each cotyledon was placed in a sterile bijou for the subsequent preparation of impression smears and the detection of chlamydial organisms by modified Ziehl–Neelsen (mZN) staining [1] and for qPCR [54]. The other half of each cotyledon was placed into a CellPath specimen container pre-filled with 10% neutral-buffered formalin (BF) (Fisher Scientific UK Ltd., Loughborough, UK; #13191184) for routine histological examination and immunohistochemistry (IHC) for confirming OEA. Three vaginal swabs (Technical Service Consultants™ Hygiene Swab; Fisher Scientific UK Ltd.; #12749945) were taken from each animal at parturition following the expulsion of the placenta for analysis by qPCR to estimate chlamydial load as a measure of bacterial shedding [54] and also by mZN when the placenta was not recovered. For any foetuses recovered from unusual or suspected non-chlamydial causes of foetal death, samples of brain, lung, heart, and liver were placed in 10% BF for histopathological investigation and IHC, to principally discount other causes of death by common abortifacient bacterial pathogens or confirm lambing issues, such as suffocation resulting from dystocia [41]. Blood samples (10 mL) were collected via jugular venipuncture into BD Vacutainer^®^ serum tubes (Fisher Scientific UK Ltd.; #12957686) prior to vaccination and at regular intervals throughout the study for serological analysis by ELISA, while an additional 20 mL blood was collected into BD Vacutainer^®^ heparin plasma tubes (Fisher Scientific UK Ltd.; #13171543) for cellular analyses (see Figure 1).

### 2.7. Quantitative Real-Time PCR (qPCR)

Two vaginal swabs were collected from each animal after expulsion of the placenta(s). These were placed into a sterile bijou and stored at −20 °C until further use. Subsequently, swabs were thawed and 1 mL sterile PBS was added to each bijou and vortexed for 30 s. The liquid was transferred to sterile microcentrifuge tubes and centrifuged at 12,500× *g* for 15 min in a standard bench-top microcentrifuge. DNA was extracted from the pellet using a DNeasy^®^ Blood and Tissue Kit (Qiagen Ltd., Crawley, UK; #69504) and eluted in 200 μL of supplied buffer AE, following the manufacturer’s instructions. Quantitative real-time PCR was carried out on all extracted swab DNA samples using primers and probes based on the *C. abortus* OmpA gene (Accession Number CR848038, gene CAB048), as described previously [54]. Briefly, the PCR reaction consisted of 2X TaqMan^®^ universal PCR master mix (Thermo Fisher Scientific (Applied Biosystems); #4364340), OmpA forward primer (5′-CGGCATTCAACCTCGTT-3′) and reverse primer (5′-CCTTGAGTGATGCCTACATTGG-3′), dual-labelled fluorescent probe (5′ FAM-GTTAAAGGATCCTCCATAGCAGCTGATCAG-TAMRA 3′; Eurofins Genomics (MWG Biotech GmbH), Ebersberg, Germany), and 1 μL of DNA and sterile nuclease-free water (Promega UK; P1193) up to a final volume of 25 μL per sample. The thermal cycling conditions were 50 °C for 2 min; 95 °C for 10 min; 45 cycles of 95 °C for 15 s; and finally, 60 °C for 1 min. Amplification and detection were performed using an ABI7000 real-time PCR system (Thermo Fisher Scientific (Applied Biosystems)), following the manufacturer’s standard protocols. Each sample was tested in triplicate and quantified against a standard curve (established with 10-fold concentrations ranging from 10^7^ to 10^1^ genome copies of *C. abortus* strain S26/3 per reaction). The results were expressed as the number of *C. abortus* genome copies per 1 uL total swab-extracted DNA. Genome copies above 100 were considered positive. In an attempt to control for environmental contamination in the post-mortem area of the farm where sampling occurs and to provide enough data to smooth out any potential day-to-day variation, this cut-off was estimated from qPCR data collected from negative control animals from all our pathogenesis studies conducted over a 15-year period. Specifically, it was determined by the upper limit of the ordinary 95% confidence interval of the mean to allow for any uncertainty (i.e., sample mean plus two times the standard error of the mean).

### 2.8. Histopathological Examination and Immunohistochemical Analysis

Placental and foetal tissues, fixed in 10% BF, were processed and embedded in paraffin wax for histopathological and IHC analysis, as described previously [58]. For histopathological examination, 5 μm serial sections were cut and stained with haematoxylin and eosin. Sections were labelled for IHC with a mouse monoclonal antibody (mAb) to the lipopolysaccharide (LPS) of *C. abortus* strain S26/3 (mAb 13/4; Santa Cruz Biotechnology, Inc., Heidelberg, Germany; #sc-101593). Bound antibodies were detected and visualised using a goat anti-mouse IgG conjugate (Dako EnVision™+ System HRP-labelled polymer (mouse); Agilent Technologies Denmark ApS, Glostrup, Denmark; #K4001), counterstained with haematoxylin, and mounted, as described previously [58].

### 2.9. Serological and Cellular Analyses

Serum samples were prepared from the blood that was collected from all animals prior to vaccination and throughout the course of the study and analysed by indirect rOMP90B-3 ELISA, as previously described [56]. Optical densities were normalised using positive and negative control sera and expressed as a percentage of the positive control using the following formula: [(OD sample-OD negative control)/(OD positive control-OD negative control)] × 100, as previously described [56].

Ovine peripheral blood mononuclear cells (PBMCs) were isolated from heparinised whole blood, counted, adjusted to 2 × 10^6^ cells/mL in complete Iscove’s Modified Dulbecco’s Medium (IMDM) and cultured in 96-well sterile U-bottom plates (Thermo Fisher Scientific (Nunc^TM^); #168136), for 96 h, as previously described [41,45]. A total of 100 μL each of purified *C. abortus* EB antigen (1 µg/mL), purified *C. abortus* COMC antigen (0.5 μg/mL), ConA (5 μg/mL; concanavalin A from *Canavalia ensiformis*, Merck Life Science UK Limited, Dorset, UK; C0412), and medium alone were added to the cells in quadruplicate wells for each animal and time point. Antigen-specific recall responses were assessed together alongside assay stimulation controls by analysis of the culture supernatants collected after 96 h for cytokines interferon-gamma (IFN-γ), interleukin (IL)-4, and IL-10, as described previously [45].

### 2.10. Statistical Analyses

Statistical modelling and associated comparisons were conducted on the R system for statistical computing version 4.4.1 [59].

Abortion and gross infection data were analysed using binomial generalised linear models (GLMs), with a logit link function and using the treatment group as an explanatory factor. The models were fitted by the maximum likelihood method, including a bias-reduction correction [60], which allowed the absence of negative cases in the challenge control group to be taken into consideration. The overall statistical significance of the group effect was assessed using the chi-square statistic.

A summary of the vaginal swab qPCR data was provided by computing geometric means and geometric standard errors of the means (SEMs), thus accounting for their multiplicative scale. The qPCR loads of vaccinated groups were compared with the challenge control group using Dunnett’s contrasts. Moreover, comparisons between vaccinated groups were conducted using Tukey’s contrasts [61].

Differences in serological and cytokine responses over bleeds were investigated using ordinary linear mixed models (LMMs) fitted by restricted maximum likelihood to rank-based inverse normal transformed data. The treatment group, bleed, and a potential interaction between them were considered in the models as explanatory factors, with the animal ID specified as a random effect. An analogous LMM approach was used when comparing lambed and aborted animals. The statistical significance of LMM coefficients relied on conditional F-tests. Post hoc pairwise comparisons were based on t-tests.

Whenever multiple statistical comparisons over treatment groups or bleeds were performed, the resulting *p*-values were adjusted to deal with type I error inflation using the Benjamini–Hochberg method, controlling for false discovery rates [62]. Statistical significance was concluded at the ordinary 5% level.

## 3. Results

### 3.1. Pregnancy Outcome

Following vaccination, animal behaviour and clinical signs were monitored, with no adverse reactions observed, which was consistent with our previous studies [35,41]. Similarly, we did not observe or record any adverse response to the challenge. The pregnancy outcomes for each of the experimental vaccine (groups 1–7), challenge control (group 8), and negative control (group 9) groups are summarised in Table 1 and detailed in Table S1. No abortions occurred in any of the animals in the negative control group 9, with all lambs delivered at the expected time with a mean gestational average of 147.2 days (range of 145–149 days; Table S1), which is in keeping with the expected average length of gestation of 147 days for sheep (typical range of 142–152 days). The majority of ewes in the experimental vaccine groups (128 of 136 ewes) delivered healthy live lambs with a mean gestational length of 144.5 days (Table S1), which is slightly lower than the mean observed in the negative control group and slightly greater than that observed in the challenge control group (average of 142.7 days; Table 1).

Single abortion events occurred in three of the vaccinated groups (groups 1 (one set of triplets), 4 (one set of twins), and 6 (one set of twins)), while two abortion events (two individuals, including a weak lamb that died within 48 h of birth) occurred in the vaccinated group receiving the 5 μg dose of COMC antigen (group 5), and three abortion events (two sets of twins and one individual) occurred in the vaccinated group receiving the lowest dose of COMC antigen (2.5 μg; group 7). These aborted animals had a wide mean gestational range of 132–150 (mean average of 139 days; Table S1), which was similar to that observed in challenge control group 8 (range of 129–147 days; mean average of 136.3; Table S1). In this challenge control group, six of the eighteen ewes aborted nine lambs (three sets of twins and three individuals), which resulted in the overall lower mean gestational length for this group. Although some abortions occurred in the vaccinated groups, the overall abortion rate was statistically significantly lower in these groups compared to the challenge control group (*p* = 0.0015), although this was largely driven by differences with groups 1–4 and 6).

A further five lambs were found dead, three of which were suspected cases of dystocia (one each in groups 5, 6, and 8), while a sixth was euthanised on humane grounds (group 3), and all of which showed no bacteriological or pathological evidence of OEA (Table 1 and Table S1).

### 3.2. Detection of C. abortus Infection

A total of 251 of 258 placentas were recovered from the ewes following lambing or abortion. Each of these placentas was examined for evidence of gross pathology typically associated with OEA. As generally seen with this disease, gross lesions were observed on the placentas of ewes that delivered apparently normal healthy lambs as well as those that aborted (Table 2). The extent of this gross pathology was largely greatest for the placentas associated with aborted lambs, where the lesions covered most of the placental surface (70–100%), although there were a number of exceptions where coverage was much lower (15–60%) (Table S1). Overall, gross pathology was much less evident in the placentas associated with live lambs (mostly 0%), but we did note that there was a relatively higher percentage of gross pathology in a number of placentas associated with live lambs in the lower vaccine dose groups (groups 6 and 7; one placenta in each group with 40 and 60% gross pathology, respectively) and even higher in the challenge control group (group 8; five placentas with 50–80% gross pathology) (Table 2 and Table S1). The results of the placental smear analyses were very similar to the gross pathology observations, with organisms detected in a few additional placentas from lambed animals where lesions were not apparent (one extra in each group of groups 1, 6, and 8 and three extra in group 7) (Table 2 and Table S1). Smear results perfectly matched gross pathology results for all placentas from aborted animals. Similarly, qPCR analysis of post-partum vaginal fluids added a further increase in the sensitivity of pathogen detection (herein referred to as ‘bacterial load’), revealing a large increase in the number of lambed animals that were deemed positive (Table 2 and Table S1). This was particularly evident in the challenge control group where the swab material from all lambed animals was found to be positive. The increase in positive animals observed in these groups is reflected in the geometric means that are considerably larger in the vaccinated groups (190–1452 *C. abortus* genome copies) and challenge control group (77,531 genome copies) than those observed in the negative control group (24 genome copies). Furthermore, the much larger geometric mean for the lambed challenge control group compared to the lambed vaccinated groups is evident from the larger number of positive animals in this group and their overall larger bacterial loads (Table S1). As would be expected, the geometric means of the aborted animals are considerably larger than for the lambed animals, and although this is reflected in the fact that all aborted animals have very high bacterial loads (7.2 × 10^5^–1.9 × 10^7^ *C. abortus* genome copies), it should nonetheless be noted that equivalent extremely high bacterial loads were observed in some individual lambed animals across all vaccinated (1.1 × 10^3^–5.5 × 10^6^) and challenge (1.6 × 10^3^–9.7 × 10^6^) groups (Table S1). Despite these high numbers, a statistically significant reduction in bacterial load in all vaccinated groups compared to the challenge control group (*p* < 0.0052) was observed. No statistically significant differences were identified between vaccinated groups 1–7 (*p* > 0.2115). Overall, the infection rate (taking into account placental gross pathology, the presence of organisms in placental smears, and the presence of bacterial DNA on post-partum vaginal swabs) was statistically significantly higher for the challenge control group compared to the vaccinated groups (*p* = 0.0037). However, this appeared to be fundamentally driven by groups 1–6, and the infection rates between the vaccinated group 7 and the challenge control group were not statistically distinguishable (*p* = 0.6331).

### 3.3. Histology and Immunohistochemical Analysis

Histological and IHC analysis was performed on a random selection of placentas and foetuses from each of the groups, principally to confirm OEA as a cause of abortion, or on placentas and foetuses resulting from the occurrence of unusual events to discount OEA as a cause, including unexpected deaths and suspected cases of asphyxiation. Analysed samples from all the abortion cases in both vaccinated and challenge control groups exhibited pathology that is indistinguishable from that which has been previously reported for this disease and is thus diagnostic of *C. abortus* infection [43,44,58,63]. This was typified by histology revealing suppurative necrotising placentitis with vasculitis in infected placentas and positive labelling for *C. abortus* antigen by IHC. For the lambs found dead in groups 1 and 2, the lamb euthanised on welfare grounds in group 2, the lamb that died after 48 h in group 5, and the lambs in groups 5, 6, and 8 that died as a result of suspected suffocation (see Section 3.1 and Table 1), histology and IHC revealed no lesions or chlamydial antigen labelling associated with OEA in any of the tissue samples examined. In contrast, for the weak lamb in group 5 that died within 48 h in group 5, the tissues examined revealed histological changes and IHC antigen labelling consistent with *C. abortus* infection.

### 3.4. Serological Responses

**Figure 2:**
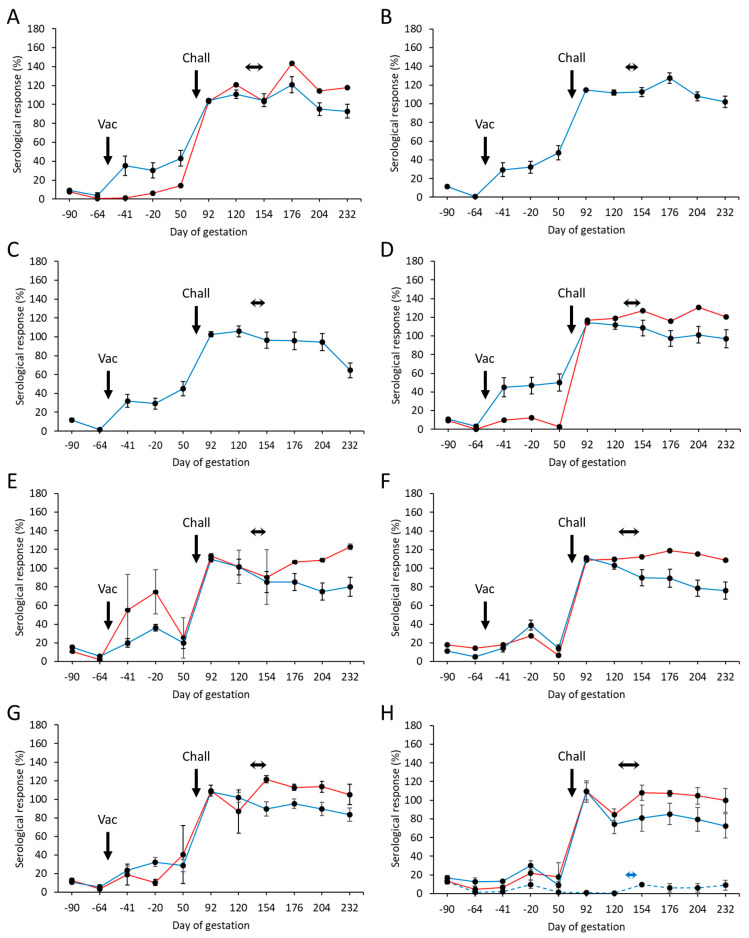
Serological responses following COMC vaccination and *C. abortus* challenge. Detection of *C. abortus* antibody in ewes vaccinated (56 days prior to mating) with a single dose (Vac) of the experimental COMC antigen preparation (20 μg (panel (**A**)), 14 μg (**B**), 10 μg (**C**), 7 μg (**D**), 5 μg (**E**), 3.5 μg (**F**), and 2.5 μg (**G**)) and challenged (Chall) on day 70 of gestation with *C. abortus* strain S26/3. Unvaccinated challenged (H; solid lines) and unvaccinated non-challenged ((**H**); dotted line) ewes served as positive and negative control groups. Data are separated into lambed (blue lines) versus aborted (red lines). Data points represent the arithmetic mean values for each cellular bleed and error bars represent the standard error of that mean (SEM). A value of 100% is equivalent to an OD450 nm of 2.25. The lambing/abortion period for each group is indicated by the horizontal black (vaccinated and challenge control groups) and blue (negative control group) double-headed arrows.

A total of 194 animals, sourced from OEA-free certified flocks, were pre-screened for *C. abortus* antibodies on two occasions prior to vaccination (Figure 1) by rOMP90B-3 ELISA [56]. One hundred and seventy-eight were selected for the study on the basis of being seronegative and having low interferon-γ responses to chlamydial antigens (see Section 3.5). Specific mean humoral responses, detected for the aborted and lambed animals in each of the vaccinated challenged groups and control groups, are presented in Table S2 and summarised in Figure 2. Responses to vaccination were detectable after 2 weeks in the four highest dose groups (Figure 2A–D) for the ewes that lambed, where the increases in titre were very similar with no significant difference in the magnitude of the responses (*p* > 0.7705). The exceptions to this were for the single aborting ewes in groups 1 and 4 where there appeared to be no or little response to vaccination, and which remained essentially serologically negative until after the challenge. Antibody responses to vaccination in only the lambed animals in the three lowest dose groups (Figure 2E–G) were lower and more delayed than for the higher dose groups, peaking around 21 days later. The responses in these lower dose groups also appeared to be more transient in nature and returned to baseline levels approximately 4 weeks later. We also noted an unexplained but similar small transient rise in antibody response 13 weeks prior to the challenge date in the control animals.

The antibody responses in the aborted animals in group 5 appeared to rise considerably following vaccination, but this result was driven by the results for one of the two ewes, hence the large error bars. Overall, by bleed 5 (pre-challenge bleed on day 50 of gestation), the antibody titres in groups 1–3 were statistically significantly higher than observed in the other vaccinated groups (*p* < 0.0232). Group 4 would have also been statistically significantly different as well if the aborted animal data were excluded from the analysis.

A rapid increase in antibody titre was observed three weeks following the challenge where responses in all vaccinated groups (Figure 2A–G) and the challenge control group (Figure 2H) peaked at a similar level. Following parturition, a difference in responses between lambed versus aborted animals was observed, where those that aborted either continued to increase in titre or remained elevated (*p* = 0.0234). Antibody titres of lambed animals generally waned, albeit slowly, after parturition for the duration of the study. All animals in the negative control group remained serologically negative throughout the study (Figure 2H).

### 3.5. Cellular Responses

The cohort of 194 sheep was pre-screened for cellular recall responses to *C. abortus* antigens (COMC vaccine antigen and EBs) and the T cell mitogen ConA on two occasions prior to vaccination (Figure 1). As part of the selection of the final cohort, animals with high IFN-γ responses to the medium alone and *C. abortus* antigens, and/or poor responses to ConA, were excluded. The final selected 178 animals were also confirmed as serologically negative as described in Section 3.4. The identified sheep were randomly split into groups assigned on the basis of similar proportions of animals with a range of lower, medium, and higher IFN-γ responses to ConA as we have undertaken for previous studies [45].

**Figure 3:**
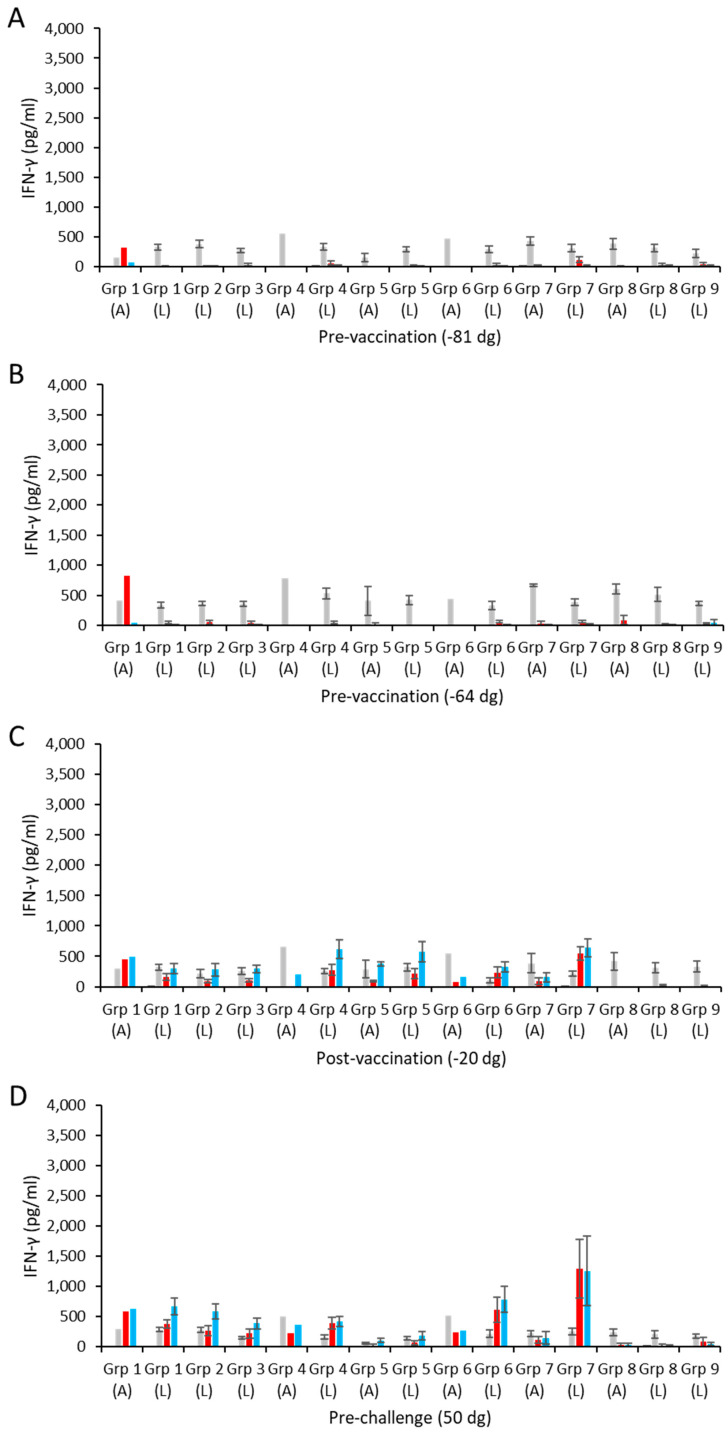

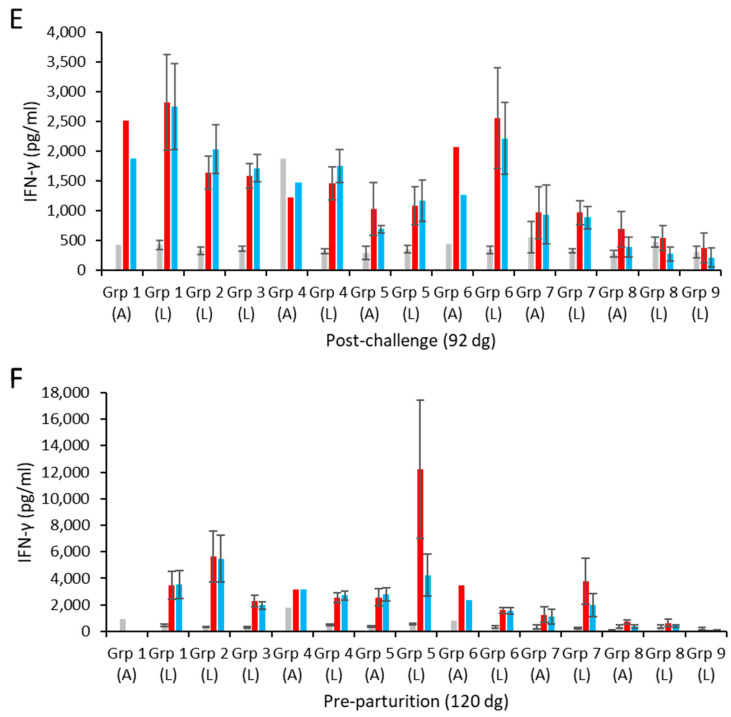
Interferon-γ responses following vaccination and challenge with *C. abortus*. Peripheral blood mononuclear cells (PBMCs) from the vaccinated, challenge control, and negative control groups were purified from whole blood (as described in Section 2.9) collected pre-vaccination (panels **A**,**B**), post-vaccination (**C**), pre-challenge (**D**), post-challenge (**E**), and pre-parturition (**F**) at the indicated time points related to the day of mating (also see Figure 1). PBMCs were set up in lymphocyte stimulation assays in vitro using the medium only as an unstimulated cell control (black bars), the mitogen concanavalin A (ConA) as a positive control (grey bars), and UV-inactivated *C. abortus* EB antigen (red bars) and COMC (vaccine antigen; blue bars) for measuring chlamydial antigen-specific stimulation. Antigen-specific IFN-γ recall responses were assessed by analysis of culture supernatants. Data points represent the mean values for each cellular bleed and error bars represent the standard error of that mean (SEM). Note the different scale for the ordinate axis in panel F.

Specific mean cellular IFN-γ responses for the aborted and lambed animals in each of the vaccinated challenged groups and control groups are shown in Figure 3 (raw data are shown in Table S2). The IFN-γ responses to ConA pre-bleeds/pre-vaccination across groups were broadly consistent (Figure 3A,B; *p* = 0.695), with only a few animals having responses to EB antigens but not to COMC or media alone. The responses to media alone and to the ConA mitogen remained consistent throughout the study (Figure 3A–F). The single animal in group 1 that went on to abort was noted to have some pre-existing cellular responses to the chlamydial antigens (Figure 3A,B; Table S2). Following immunisation, vaccine antigen-specific (COMC) IFN-γ responses were observed across all of the vaccine groups (groups 1–7; Figure 3C) at levels equivalent to or greater than the positive mitogen control ConA. The responses in the pre-challenge pregnant sheep (day 50 of gestation) appear to be more variable within the vaccinated groups, indicating subtle variability between lambed and aborted animals. Broadly, there was good consistency in IFN-γ responses to both COMC and *C. abortus* EBs (*p* > 0.0514). However, we noted that IFN-γ production in group 5 was significantly lower than in the other groups at this point (*p* < 0.0297) (Figure 3D).

Following the experimental challenge, a strong up-regulation of *Chlamydia*-specific IFN-γ responses across all of the vaccinated challenged groups (*p* < 0.0019) was observed, with the smallest change noted for group 7 (*p* = 0.0439) (Figure 3E). In general, this up-regulation of IFN-γ was broadly similar in magnitude across these vaccinated groups (excluding group 7). Although some of the animals that aborted had higher antigen-driven responses than many of those that lambed (day 92 of gestation in Table S2), they were not found to be statistically significantly higher (*p* = 0.9903), principally because, overall, the lambed responses were highly variable. Some low responses were observed in the negative control group (group 9); however, these responses were generally negligible (Table S2) and close to the limits of detection for the ELISA test.

In the final cellular bleed prior to parturition (Figure 3F), the responses are much greater across the vaccinated challenge groups (Figure 3F; groups 1–7; note change in scale for the ordinate axis) compared to earlier time points (Figure 3C–E) with no significant differences (*p* = 0.1010) in *Chlamydia*-specific IFN-γ responses. The exception to this was for the lambed animals in group 7 (Figure 3F), where there was a lower response to the COMC antigen (*p* < 0.0159).

**Figure 4:**
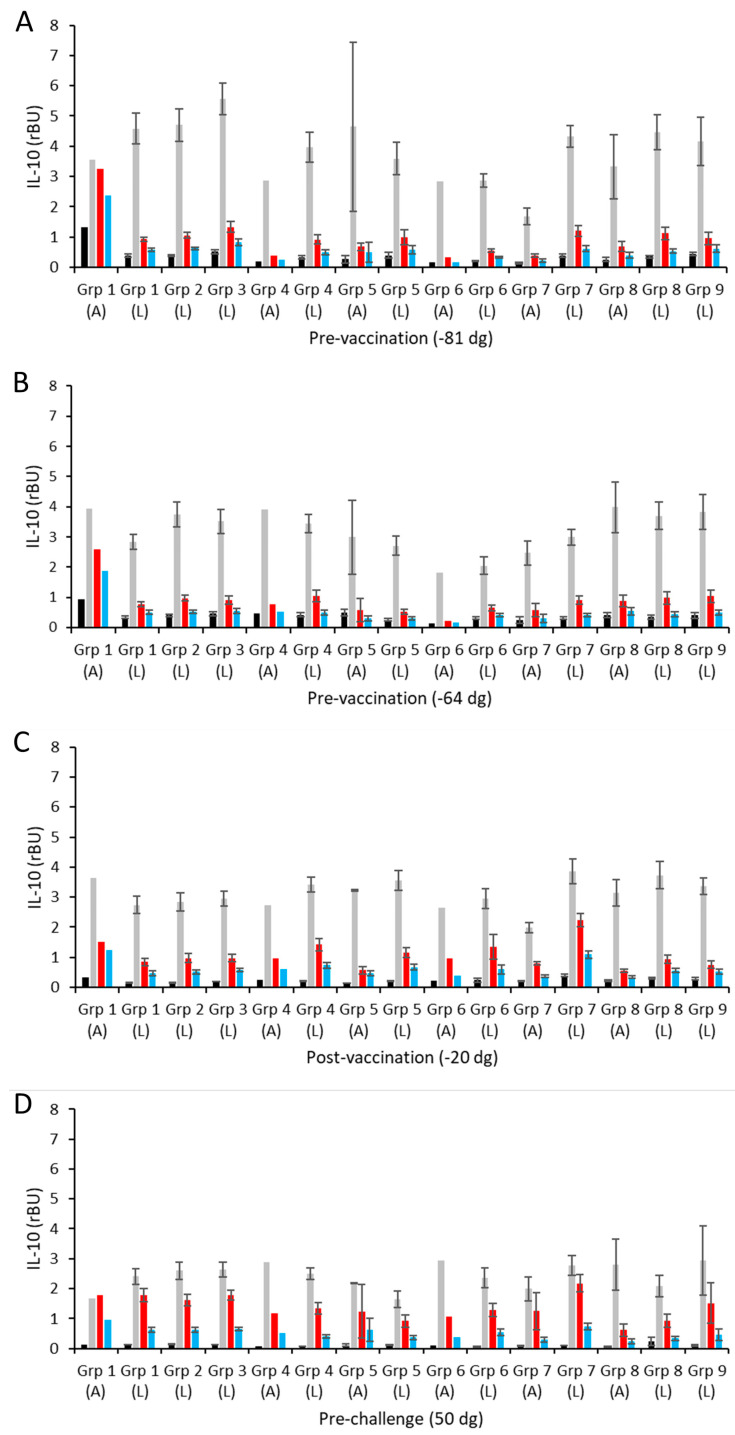

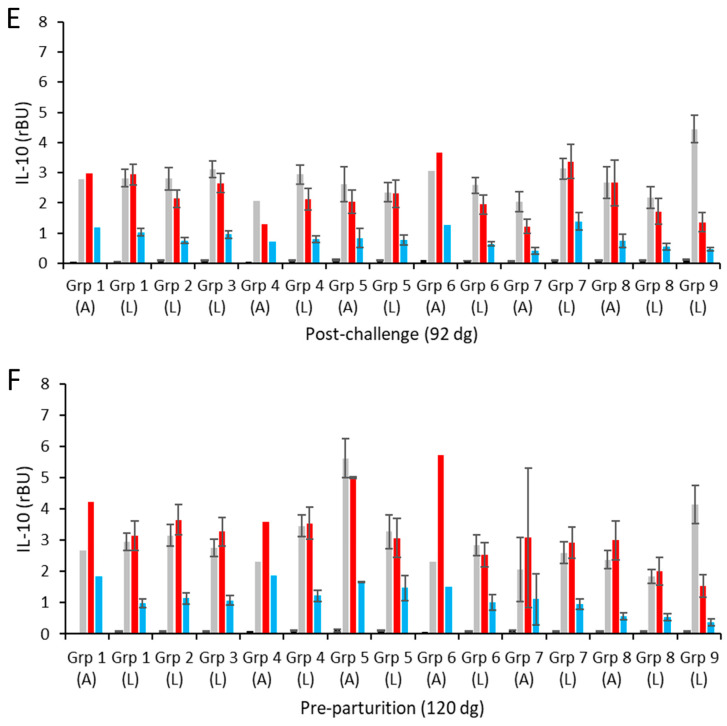
IL-10 responses following vaccination and challenge with *C. abortus*. Peripheral blood mononuclear cells (PBMCs) from the vaccinated, challenge control, and negative control groups were purified from whole blood (as described in Section 2.9) collected pre-vaccination (panels **A**,**B**), post-vaccination (**C**), pre-challenge (**D**), post-challenge (**E**), and pre-parturition (**F**) at the indicated time points in relation to the day of mating (also see Figure 1). PBMCs were set up in lymphocyte stimulation assays in vitro using the medium only as an unstimulated cell control (black bars), the mitogen concanavalin A (ConA) as a positive control (grey bars), and UV-inactivated *C. abortus* EB antigen (red bars) and COMC (vaccine antigen; blue bars) for measuring chlamydial antigen-specific stimulation. Antigen-specific IL-10 recall responses were assessed by analysis of the culture supernatants. Data points represent the mean values for each cellular bleed and error bars represent the standard error of that mean (SEM).

Supernatants from antigen-/mitogen-/medium-stimulated PBMCs from each of the experimental vaccine and control groups were also screened for IL-10 responses. The resulting mean IL-10 responses for the aborted and lambed animals for each group are shown in Figure 4 (raw data are shown in Table S2). In both pre-vaccination bleeds (Figure 4A,B), the IL-10 responses are as expected with strong consistent responses between groups to ConA stimulation and no or negligible responses to media and the chlamydial antigens, mostly at levels around the limit of sensitivity of the ELISA. The intra-group IL-10 responses seemed quite variable to ConA (Figure 4A,B; Table S2). As with the IFN-γ response, the single aborted ewe in group 1 had elevated antigen-specific IL-10 (Figure 4A,B), although this difference was no longer evident post-vaccination. The ConA responses across vaccine challenge and control groups were consistent across all sampling time points (Figure 4A–F) (*p* = 0.1387). Responses to media alone were generally below the ELISA sensitivity threshold and hence are not evident in Figure 4.

Following vaccination and pre-challenge, there was no marked increase in chlamydial antigen-driven IL-10 production (Figure 4C,D) as mean values appear to be within a two-fold magnitude of the responses in the non-vaccinated control groups (groups 8 and 9) (*p* = 0.3584). However, the responses to the *C. abortus* EBs notionally appear a little higher than to the vaccine antigen (COMC) across the groups (Figure 4C,D). Indeed, this difference between EB- and COMC-driven responses was much greater post-challenge and pre-parturition (Figure 3E,F) (*p* < 0.0001), where the EB antigen-driven IL-10 responses were elevated to levels approaching (Figure 4E) and exceeding (Figure 4F) the ConA mitogen control levels (Figure 4E,F). There was no significant association between the magnitude of responses to chlamydial antigens and the administered vaccine antigen dose and no apparent increase in IL10 production between post-challenge (Figure 4E) and pre-parturition bleeds (Figure 4F) across all the groups (*p* = 0.0966).

In contrast to the cellular IFN-γ and IL-10 production data, there was no evidence of any counter-regulatory IL-4 production to vaccination. PBMC responses as expected were low toward the medium alone and high toward the T cell mitogen ConA.

## 4. Discussion

Here, we report further optimisation of an experimental subcellular vaccine based on a sarkosyl-extracted outer membrane preparation of *C. abortus*, known as the COMC [36]. Previous studies have compared the protective efficacy of the vaccine, delivered in two 10 μg doses three weeks apart, to another experimental vaccine and to the commercial live-attenuated vaccine Cevac^®^ Chlamydia (Ceva Animal Health Ltd., Amersham, UK) where it was shown to be highly effective in reducing abortions and the shedding of infectious organisms post-parturition [35]. A follow-up study showed that the vaccine could be delivered as a single inoculation of 20 μg and that this dose could be halved without compromising its efficacy in reducing the number of abortions [41]. In this present investigation, we have extended these studies to further optimise the dose of antigen that could be used in a commercial vaccine formulation by conducting a dose–response experiment, where we evaluated a range of antigen doses decreasing from 20 to 2.5 μg, using our well-established pregnant sheep challenge model. We have shown in many of our pathogenesis studies over the last four decades that this model mimics a natural field infection and is highly reproducible [16,35,43,45,46], and it is also used by other groups [18,64]. The COMC antigen preparations were again formulated using the adjuvant Montanide^TM^ ISA 70 VG that we used in the previous studies and which we found to be effective in driving strong chlamydial antigen-specific IFN-γ recall responses [35,41].

Initial evaluation of vaccine efficacy was based on the simple readout of clinical outcome in each experimental vaccine group compared to the challenge control. Although we observed no abortions in two of the higher dose vaccine groups (14 and 10 μg), we did observe single abortion events in three of the experimental vaccine groups, including the highest dose group (doses of 20, 7, and 3.5 μg), while two of the lowest dose groups had two (5 μg) and three (2.5 μg) abortions. The abortion of triplets in the highest dose group was a little surprising considering the positive results we had observed in our previous two pregnant sheep vaccine trials with this dose, where no abortions had occurred [35,41]. We are not sure of the reason for this, but it may relate to having used gimmers (also known as theaves; names of young female sheep before their first lambing) in this study compared to older sheep in the previous studies, and, in general, these younger animals may be more susceptible to infection by the pathogen due to a less mature immune system [65]. Another possibility is that this animal was not immunologically primed to respond, as suggested by the serological data where there was a minimal antibody response to vaccination. More abortions occurred in the lowest dose groups (less than 10 μg), as might be expected. However, we did not observe any gradual increase in the number of abortions as the dose of antigen was decreased, although the lowest dose did produce the greatest number of abortions, but even then, this was still significantly less than that observed in the challenge control group. Overall, despite the abortions occurring in the vaccinated groups, there was still a statistically significant difference between them and the challenge control group, particularly for groups 1–4, showing that all of the vaccine doses were effective in reducing the number of abortions in the animals following the challenge.

The biggest sources of infection responsible for the potential transmission of infectious organisms to naïve animals are the placenta and vaginal fluids excreted from a ewe post-parturition. Therefore, we also evaluated the extent of gross placental pathology (percentage lesion coverage of the placental surface) and the presence (by mZN staining of placental smears) and organism burden or load (by qPCR of vaginal swabs) for both of these products of abortion. We consider this as additionally important to the clinical outcome when evaluating the efficacy of the experimental vaccine. Initially, we inspected every collected placenta and estimated the extent of the gross lesions covering the placental surface. The placentas from the aborted animals all showed extensive lesions (generally 50–100%), irrespective of whether they occurred in the vaccinated or the challenge control animals. This extent of lesion coverage is typical of what we observe in the field in the placentas of animals that have aborted due to OEA, which was confirmed following pathological investigation. Additionally, this qualitative assessment gave us a good indication of the level of infection in the placentas of lambed animals compared to those that aborted and revealed a small number of placentas from animals that lambed to show evidence of placental infection (18 of 171 placentas; ranging from 1 to 60% across groups), with only the group 4 (7 μg dose) placentas showing no evidence of gross placental pathology. This contrasts with the challenge control group where there was a significantly higher number of positive placentas with a greater extent of lesion coverage from the lambed animals (11 of 20 placentas; ranging from 1 to 80%). This suggested that despite the fact that some of the vaccinated lambed animals had evidence of infection, it would appear that the vaccine had nonetheless controlled the level of infection compared to the non-vaccinated challenged animals. This was further supported by the estimated lesion coverage being a lot less in the higher antigen dose groups (up to 25% across groups 1–5) than the lower dose groups (up to 40% in group 6 and 60% in group 7) or the challenge control group (up to 80%).

The results from the placental smears essentially agreed with the gross placental pathology results, revealing a small increase in the sensitivity of detection of infection, with EBs observed in nine extra samples from the placentas of the lambed animals. The qPCR results on the swab material added a further level of sensitivity with an extra sixty-nine animals considered positive, although the number of genome copies present in these samples varied quite markedly from low (<1000 copies) to very high (>10^6^ copies), the latter being comparable to levels we routinely observe in animals that have aborted. As we have mentioned above, we are not clear on why this result differs from what we have observed in our previous studies [35,41], other than the possibility of it being due to the use of younger and potentially more susceptible animals in this study. Of course, we also need to remember that these levels are based on DNA and not on infectious organisms, and previous work (unpublished observations) has estimated that the two can differ by a factor of around one hundred (with the number of live infectious organisms being 100-fold lower); therefore, despite these figures, most of the animals are not likely to have a major impact in terms of potential transmission of infectious organisms to naïve animals. This is supported by the geometric mean results for each of the groups, where we observed much lower mean genome copies for the vaccinated lambed animals compared to the lambed challenge control animals, suggesting a lower potential risk of transmission from the vaccinated lambed animals to naïve animals compared to the unvaccinated (challenge control) animals. Furthermore, although there appeared to be no clear antigen dose effect on protective efficacy in the vaccinated groups, we did observe that the highest dose group had a much greater proportion of animals (10 of 19; 52.6%) that were negative overall compared to the lowest dose group (2 of 21; 9.5%), with the middle doses being similar to each other (27.8–40.0%) but still much greater than the lowest dose group. But even the antigen in the lowest dose group had a positive effect on the infection rate, with a much lower swab qPCR geometric mean for the animals that lambed (1030 genome copies) compared to those in the unvaccinated challenge control group (77,531 copies), where all the lambed animals were deemed positive. In agreement with the gross placental lesions and placental smear scores for the animals that aborted in the vaccinated and challenge control groups, there was essentially no difference in the number of genome copies detected from the vaginal swabs. As well as being very similar (mostly > 10^6^ genome copies), they were comparable to what we have observed previously from aborted animals [35,41,43,44]. Why these animals failed to be protected from abortion by some of the vaccine formulations is unclear. It could be due to many things, such as genetics, stress, underlying clinical issues, age, or some other factor we do not know, but these are not unusual events, with many veterinary vaccines not eliciting 100% protection. Indeed, we did observe that some of the vaccinated animals that aborted did not respond immunologically to vaccination and hence were not protected from abortion. However, it does not detract from the fact that the vaccines, particularly those used in groups 1–6, have successfully controlled the level of shed in organisms post-parturition compared to the non-vaccinated challenged animals, significantly lowering the potential risk of transmission of infection to other naive animals.

Antibody responses were more elevated following vaccination in groups 1–4 compared to groups 5–7. The responses in groups 5–7 appeared to rise more gradually but then dipped prior to the challenge; however, we also observed a similar response to this in the challenge and negative control animals. We are not sure of the reason for this, but it does appear to be an anomaly that may affect all the samples analysed at this time point (three weeks prior to mating). Regardless of this, the responses to vaccination in groups 1–4 were statistically significantly higher than in the other challenged groups but not statistically significantly different from each other. We also noted that the responses to vaccination were not as high as those observed in the previous two studies [35,41]. Again, we are unsure of the reason for this, but while it could in part be due to a less mature immune system in the younger animals [65] used in this study, the age of the animals alone is unlikely to be the sole factor. The lack of prior microbial or pathogen exposure may also be a contributory factor as could host genetics, impacting resistance or susceptibility to infection and by extension host immunity [66]. Vaccine-induced immune responses and protection of sheep against the gastrointestinal nematode *Teladorsagia circumcincta* have been shown to be affected by age and breed [67,68,69].

Following the challenge, all vaccinated groups showed statistically similar antibody profiles, with no clear dose effect, and similar profiles to the unvaccinated challenge control group. However, in contrast to what we have observed before, where the challenge control response is usually lower than observed for the vaccinated groups, in this study, there was no statistical difference between the level of antibody response between the vaccinated and challenge control groups. This could suggest that the vaccination has not primed the immune response as well prior to the challenge, but this does not seem likely given that vaccinated animals were clearly protected from abortion compared to the challenge group and had reduced placental pathology and reduced bacterial loads post-parturition. For the animals that aborted, we saw a greater antibody response than in the lambed animals post-abortion/lambing, which is very similar to what we have observed previously [35,41]. This supports the view that antibodies do not actually have a major role in restricting the placental infection that ultimately results in the abortions occurring and are instead a response to increased antigenic stimulation that occurs as the organisms rapidly multiply in the placentas of animals that have not been protected by the vaccination. This also agrees with the view that cellular responses are more important than humoral responses for controlling chlamydial *C. abortus* infections [70,71]. Indeed, the pro-inflammatory cytokine IFN-γ has long been known to be important in restricting chlamydial growth in vitro, thus controlling infection [71,72]. For this reason, we also monitored IFN-γ responses at key stages following vaccination and during pregnancy, during which we observed broadly similar chlamydial antigen (EB and COMC)-driven responses in all vaccinated groups compared to the controls. We did note an elevated response to EB and COMC antigens for the group 1 aborted animal, which might be due to some prior exposure to a close antigenically related pathogen. Following the challenge up to parturition, the responses were found to be much greater, consistent with the animals being primed by the vaccines, and although there was no significant difference between most of the groups, a statistically significant lower response was observed for the lowest dose (group 7) animals, demonstrating no clear dose effect. The reason for the larger increase in EB compared to COMC antigens is unclear but could perhaps be due to cross-reaction with another chlamydial pathogen or other pathogens with similar outer surface antigens, where the cross-reaction is with an antigen that is lost during the extraction of the COMC from whole *C. abortus* EBs.

The effectiveness of the pro-inflammatory cellular IFN-γ responses to limit chlamydial growth can be restricted by the counter-regulatory cytokines IL-10 and IL-4 within the same cultures [73]. Analysis of samples revealed that there was no IL-4 production, while IL-10 production was evident but low and fairly consistent across all vaccine groups, albeit not significantly different from the levels observed in the control groups. These levels were not particularly elevated post-challenge or just prior to parturition, although we did note a larger difference in the response to EB compared to COMC antigens which occurred in both the vaccinated and control groups. These results may be reflective of the greater antigenic diversity in the EBs than the extracted COMCs, showing differences between responses to *Chlamydia* versus just the COMC vaccine antigen, which we are directly comparing for the first time. Here, responses to the whole EB antigen appear greater than what is derived from the COMC alone. Overall, the cellular cytokine data show that the vaccines stimulate a broad Th-1 response, and the quality of this response is unaltered by the vaccine antigen doses trialled in this study. These data for responses to EBs are nonetheless consistent with what we have observed previously [35,41]. Collectively, these analyses demonstrate the difficulties in defining individual immunological correlates of protection for chlamydial vaccine design due to the complexities of the host immune responses to *Chlamydia*, which is a common feature of chlamydial vaccine studies over the past seven decades [74].

## 5. Conclusions

In this study, we have extended our previous investigations to develop a new efficacious COMC vaccine that protects sheep from OEA by further optimisation of the vaccine dose. The vaccine has a number of advantages over the existing commercial vaccines: (1) it has no inactivation step during manufacture like for an inactivated vaccine that modifies surface antigens, which potentially affects the efficacy and protective immunity; (2) it is essentially a native antigen preparation comprising native intact outer membrane proteins and so it has all the immunological benefits of a live vaccine, without any possibility of the vaccine replicating in the host and causing infection and disease; (3) as it essentially mimics a live vaccine, it should have a long duration of immunity (although this will need to be confirmed following commercial manufacture); and (4) we have shown it is more effective than the existing commercial vaccines in limiting the shedding of the pathogen post-partum, which is important for limiting the transmission of the pathogen to other naïve animals. Vaccine efficacy was evaluated for each antigen dose by taking into account the clinical outcome, pathology, and bacterial load, as well as the humoral and cellular responses to vaccination and challenge. The combined data enabled us to determine the reduction in abortion and in the shedding of organisms and thus provide an assessment of the protective efficacy of each administered dose, as well as any associated reduction in the potential for environmental transmission of infection. Overall, there was no clear dose–response effect; instead, we found that there was little difference between the groups, other than for the lowest 2.5 μg dose. Ultimately, the finalised dose will be determined following further commercial refinement and evaluation, but our data suggest that a dose of 10 μg would build in a sufficient buffer to maximise efficacy. The final stage in our developmental pipeline for the vaccine will be to compare the adjuvant utilised in this study and the previous studies with other adjuvant formulations to see if efficacy can be further improved.

## Figures and Tables

**Table 1:** The clinical outcome of pregnancy in vaccinated ewes that were challenged with *Chlamydia abortus* strain S26/3 at day 70 of gestation (groups 1–7), non-vaccinated challenge control ewes (group 8), and uninfected non-vaccinated negative control ewes (group 9).

Group ^1^ (Dose in µg)	Ewes			Mean Gestational Length		Number of Lambs		
	No. Pregnant	No. Lambed (%)	No. Aborted (%)	Lambed	Aborted	Viable	Non-Viable	Dead
1 (20)	19	18 (94.7)	1 (5.3)	144.1	134.0	26	0	4 ^2^
2 (14)	19	19 (100)	0 (0)	145.0	-	32	0	0
3 (10)	21	21 (100)	0 (0)	144.3	-	30	1 ^3^	1 ^3^
4 (7)	20	19 (95)	1 (5)	144.3	136.0	31	0	2
5 (5)	18	16 (88.9)	2 (11.1)	144.9	139.5	25 ^4^	1 ^4^	2 ^5^
6 (3.5)	18	17 (94.4)	1 (5.6)	145.2	132.0	25	0	3 ^5^
7 (2.5)	21	18 (85.7)	3 (14.3)	143.7	143.7	31	0	5
8	18	12 (66.7)	6 (33.3)	142.7	136.3	23	0	10 ^5^
9	6	6 (100)	0 (0)	147.2	-	6	0	0

^1^ Groups 1–7, vaccinated dose of COMC is indicated in brackets; group 8, challenge controls; and group 9, negative controls. ^2^ Includes one lamb found dead, which, following bacteriological and pathological investigation, was found not to be due to OEA. ^3^ One lamb was found dead and one weak lamb was euthanised on welfare grounds shortly after birth from the same ewe, with no bacteriological or pathological evidence of OEA. ^4^ One lamb born live that died out with 48 h and one that was born weak and died within 48 h, both from the same ewe, with bacteriological and pathological evidence of OEA. ^5^ Includes the death of a single lamb in each group of groups 5, 6, and 8 due to dystocia/suffocation.

**Table 2:** Gross placental pathology, detection of *Chlamydia abortus* organisms in placental smears, and detection of genomic DNA in vaginal swabs of vaccinated ewes that were challenged with *C. abortus* at day 70 of gestation (groups 1–7), of infected control ewes (group 8), and of uninfected control ewes (group 9).

Group ^1^ (Dose in µg)	Pregnancy Outcome ^2^	No. Ewes	Lesions ^3^	Smears ^4^	Swab qPCR ^5^	Swab qPCR Load ^6^
1(20)	Lambed	18	2+, 16−	3+, 15−	8+, 10−	226 (1.96)
	Aborted	1	1+	1+	1+	n/a ^7^
2 (14)	Lambed	19	2+, 17−	5+, 14−	13+, 6−	1452 (2.47)
3 (10)	Lambed	21	2+, 19−	2+, 19−	15+, 6−	426 (1.77)
4 (7)	Lambed	19	0+, 19−	0+, 19−	11+, 8−	190 (1.90)
	Aborted	1	1+	1+	1+	n/a ^7^
5 (5)	Lambed	16	3+, 13−	3+, 13−	11+, 5−	422 (2.02)
	Aborted	2	2+	2+	2+	4,378,573 (1.37)
6 (3.5)	Lambed	17	3+, 14−	4+, 13−	12+, 5−	688 (2.51)
	Aborted	1	1+	1+	1+	n/a ^7^
7 (2.5)	Lambed	18	2+, 16−	5+, 13−	16+, 2−	1031 (2.15)
	Aborted	3	3+	3+	3+	2,157,470 (3.88)
8	Lambed	12	7+, 5−	8+, 4−	12+, 0−	77,531 (3.20)
	Aborted	6	6+	6+	6+	4,357,997 (1.65)
9	Lambed	6	6−	6−	6−	24 (1.16)

^1^ Groups 1–7, vaccinated dose of COMC is indicated in brackets; group 8, challenge controls; and group 9, negative controls. ^2^ See Table 1. ^3^ Number of ewes with gross pathological lesions characteristic of *C. abortus* infection evident in one or more placentas: +, positive; −, negative. ^4^ Number of ewes with chlamydial organisms detected following modified Ziehl–Neelsen (mZN) staining of placental smears: +, positive; −, negative. ^5^ Number of ewes with chlamydial organisms detected on vaginal swabs by quantitative real-time polymerase chain reaction (qPCR): +, positive; −, negative. ^6^ Geometric mean (geometric SEM) of the number of *C. abortus* genomes per 1 μL total DNA extracted from vaginal swabs and detected by qPCR. ^7^ n/a, not applicable (summary statistics cannot be computed for a single value).

## Data Availability

Data are contained within the article or Supplementary Materials. The original contributions presented in the study are included in the article/Supplementary Materials, and further inquiries can be directed to the corresponding author.

## Supplementary Materials

The following supporting information can be downloaded at: https://www.mdpi.com/article/10.3390/vaccines13010089/s1, Table S1: Raw data on clinical outcome, placental gross pathology, mZN, and PCR analysis for each experimental group; Table S2: Raw data for serological and cellular analyses.
